# Fresh Moringa Stenopetala leaves consumption and its determinants among pregnant women in southern Ethiopia

**DOI:** 10.3389/fnut.2024.1339819

**Published:** 2024-07-10

**Authors:** Zeritu Dewana Derbo, Gurmesa Tura Debelew

**Affiliations:** ^1^Department of Midwifery, Arba Minch Health Science College, Arba Minch, Ethiopia; ^2^Department of Population and Family Health, Institute of Health, Jimma University, Jimma, Ethiopia

**Keywords:** Moringa stenopetala, pregnant women, Gamo zone, southern Ethiopia, consumption

## Abstract

**Background:**

A woman’s health and nutritional status has significant impact on her pregnancy situation. However, many pregnant women are undernourished. Moringa stenopetala is a plant consumed worldwide in various forms, and its consumption showed a reduction in the incidence of malnutrition. Although Moringa stenopetala is rich in essential macro- and micronutrients, there is little evidence on the proportion and determinants of fresh Moringa stenopetala leaf intake among pregnant women. The objective of this study was to fill this gap in the littérature and provide a baseline evidence for further research or intervention by investigation the proportion and determinants of fresh Moringa stenopetala leaf intake among pregnant women in the Gamo zone, south Ethiopian region.

**Methods:**

A community-based cross-sectional study was conducted among 623 randomly selected pregnant women using a pre-tested and structured questionnaire via a face-to-face interview. The consumption pattern was assessed based on a self-reported dietary history over the last 30 days before data collection. Multivariable logistic regression model was fitted using STATA version 14. An adjusted odds ratio with a 95% confidence interval was reported to show an association between the dependent and independent variables with level of statistical significance at a *p*-value of <0.05.

**Results:**

The proportion of fresh Moringa stenopetala leaves intake among pregnant women was 49.60% (95% CI: 45.67, 53.52%). The déterminants of fresh moringa leaf intake were being below 24 years old (AOR: 2.92; 95% CI: 1.51, 5.63), rural résidence (AOR: 1.97; 95% CI: 1.10, 3.50), antenatal care attendance (AOR: 2.08; 95% CI: 1.03, 4.21), history of contraceptive use (AOR: 1.88; 95% CI: 1.03, 3.55), and having a good knowledge about the importance of moringa Stenopetala (AOR: 9.76; 95% CI: 5.30, 17.95).

**Conclusion:**

The study showed that almost half of the pregnant women consumed fresh Moringa stenopetala leaves. Women’s age, place of residence, prenatal care, history of contraceptive use, and knowledge of the benefits of Moringa Stenopetala were positively associated with the consumption of fresh Moringa Stenopetala leaves. Therefore, health authorities and stakeholders involved in maternal and child health need to target older women, and urban residents and promote the benefits of consumption by strengthening uptake of maternal health services and raising awareness about Moringa Stenopetela. Future studies involving large scale and longitudinal designs evidence are required to further validate the findings so that this nutritious diet can be promoted widely among pregnant women in the study area and Ethiopia at large.

## Introduction

Women have unique nutritional needs throughout their lifespan, especially during pre-pregnancy and pregnancy periods. Access to nutritious, safe, affordable, and sustainable food is crucial for women’s survival, health and well-being. Pregnancy also increases women’s need for protein, vitamins and minerals (1).

Despite this, 154 million (10%) and 520 million (33%) women worldwide were underweight and anemic, respectively, in 2020 (2). The overall pooled prevalence of malnutrition during pregnancy was 23.5% in Africa (3) and 29.07% in Ethiopia in 2021 (4). Inadequate nutritional intake is common among pregnant women due to food taboos, food insecurity, infectious diseases, and inadequate care (5–9). Malnutrition during pregnancy increases the incidence of low birth weight, preterm birth, poor APGAR (appearance, pulse, grimacing, activity, and respiration) scores, stillbirth, neonatal death, and postpartum hemorrhage, leading to poor pregnancy outcomes (10, 11). In contrast, better nutrition during pregnancy is essential to promote fetal health, survival, growth, and development. It is also a key strategy to reduce the burden of malnutrition, incidence of non - communicable diseases, and maternal and child mortality (12–18). However, most pregnant women in Ethiopia have poor and inadequate nutritional practices, with a total prevalence of 67.7% was malnourished (7, 19). Therefore, improving maternal nutritional status is an urgent priority.

Moringa Stenopetala (MS) is a fast-growing drought-resistant tropical tree that originated in India (20). This is commonly referred to as the “miracle tree,” (21) “never-dying tree,” (22) and “mother’s best friend” (23). It has different names in different localities, such as “Nébéday” in Senegal, “Yevu-ti” in Ghana and Togo, “Malunggay” in the Philippines (24), “Mlonge” in Kenya and Tanzania, “Zakalanda” in Zimbabwe, horseradish tree in the United States (24), horseradish or ben oil tree in the United Kingdom, “drumstick tree” in India (21, 22), and “Shiferaw” in Ethiopia (25). It also has different vernacular names in southern Ethiopia, such as “Aleko” or “Aluko” or “Haleko” in Gamo and Wolaita, “Kallanki” in Bena, “Telahu” in Tsemay, “Haleko” or “Shelchada” in Konso, and “Haleko” in Burje and Derashe (25).

Studies showed MS is safe for pregnant women and has a variety of nutritional and health benefits including reducing the risk of malnutrition, low birth weight, and anemia. Consuming MS during pregnancy may also help prevent DNA damage caused by stress and adverse pregnancy outcomes and improve breast milk supply. Additionally, MS has been shown to have anti-inflammatory and antioxidant effects that help fight disease**s** (26–33). Evidence also showed that MS leaves are rich in flavonoids, calcium, and antioxidants, making them suitable for consumption by pregnant women. In addition, MS leaf extract nanoparticles are reported to reduce anxiety levels among pregnant women with high blood pressure, and affected their blood pressure by increasing serotonin levels (34).

A country-wide study in Ethiopia found that 100 g of MS leaves contained 28.44% protein, 0.7% fat, 38.49% carbohydrate, 11.62% fiber, 54.85 mg iron, 1,918 mg calcium, 2.16 mg zinc, 2,094 mg potassium, 214.10 mg sodium, 28.49 mg vitamin C, 12.95 mg vitamin A, and other important nutrients (25). The finding indicate that MS species found in Ethiopia are rich in nutrients and their consumption helps in obtaining important macro- and micronutrients (25, 35–37). Current evidence in Ethiopia suggests that rising food prices will affect the intake of nutritious foods (38). MS leaves are a great way to fill this gap as they are rich in macro- and micronutrients and cheap as well.

Studies on consumption of MS species showed that it was widespread practice: 63% in India (39), 81% in Africa (39), 87.2% in Togo (40),and 90% in South Africa (41) but it has been underutilized (36%) in East Shoa Zones of Oromia,Ethiopia (42). Age, education level, marital status, occupation, household wealth status, awareness level, knowledge of plant biology, gender, and family size (43, 44) were factors associated with MS species consumption in the general population. However, such studies on the extent and determinants of MS use are lack among pregnant women. Although MS is widely cultivated in Ethiopia and has been indicated as having nutritional and health benefits for pregnant women, little is known about the level of consumption and factors affecting this during pregnancy. There are few studies on the nutritional, medicinal, and water purification properties of MS, and its effect on maternal hemoglobin levels, but there is little scientific evidence supporting MS consumption in pregnant women (25, 45). Therefore, this study aimed to investigate the level of consumption of fresh MS leaves, and factors associated with it among pregnant women in Gamo zone, southern Ethiopia.

## Materials and methods

### Study setting

The study was conducted in the Arba Minch Zuria and Chencha Districts, Gamo Zone, southern Ethiopia. It is located 434 Km and 443 km south of Addis Ababa (the capital city of Ethiopia) respectively (46). Based on projection made from the 2007 Ethiopian population and housing census the population residing in the two districts summed up to 353,019 in 2021/22, and the expected number of pregnant women was estimated to be 12,214. The area has two hospitals, nine health centers, sixty-three health posts, and a number of private health facilities at different levels of service standards that collectively provide curative, preventive, and rehabilitative services for the population (45).In the study area, fresh MS leaves are commonly prepared and consumed in the form of ‘Kurkufa’, and ‘Fosossie’ (through directly cooking a corn flour or its wraps with the leaves), and ‘Kita’(in the form ofa flat bread locally prepared from various cereals including corn, wheat, barley or ‘Teff’), and ‘Haleko’ (directly cooking the MS leaves in a separate dish like spinach and/or in the form of soup).

### Study design and period

A community-based cross-sectional study was conducted from May 8th to June 20, 2022.

### Population of the study

The source populations were all pregnant women living in Arba Minch Zuria and Chencha district, and the study populations were pregnant women in selected kebeles (*smallest administrative unit in Ethiopia*) in Arba Minch Zuria and Chencha district.

### Eligibility

#### Inclusion criteria

All pregnant women who were in between 20 and 26 weeks of gestation at the time of recruitment and permanently residing in Arba Minch Zuria and Chencha district were included in the study.

#### Exclusion criteria

Women who did not give consent to participate in the study and who were seriously ill or unable to give information for the interview were excluded from the study. Women were considered as seriously ill when they had a health condition that made them unable to speak and carried a high risk of mortality and were more generally not able to perform activities and daily living and were excessively straining their care givers (47).

### Sample size and sampling procedure

The sample size required to assess fresh MS consumption habits in pregnant women was estimated using the sample size estimation formula for single population proportions using Epiinfo version 7.2.3.1. As there are no previous studies on the proportion of fresh MS leaf consumption habits among pregnant women, we assumed a proportion of fresh MS leaf consumption during pregnancy (50%) (48, 49), and a confidence level of 95%, a margin of error of 5%. Considering the design effect of 1.5 for compensation of potential error due to the multistage cluster sampling and estimated non-response rate of 10%, the final sample size was 633.6(634).

A multistage cluster sampling method was used to recruit study participants into the study. From zone two districts were included in the survey. First, 10 kebeles from each of the districts were selected by lottery method. The list of pregnant women was taken from family folders available at the health posts, and samples were proportionally allocated to each of the selected kebeles based on the number of pregnant women with gestational age between 20 and 26 weeks. Finally, the required pregnant women were selected using simple random sampling technique from the sampling frame of pregnant women prepared for each kebele.

### Study variable

The dependent variable was consumption of fresh MS leaves and the independent variables were socio-economic and demographic factors (age of mother, Place of residence, Educational Status), health and obstetric related factors (Gravidity, Number of <5 children, Pre- Contraceptive use, ANC attendance), nutritional factors (Changed dietary intake, Dietary diversity, Food aversion,) and knowledge about MS.

### Operational definitions

MS consumers: women who consumed fresh moringa leaves at least once in the last 30 days (one month) before the data collection date regardless of amount or frequency were classified as consumers; otherwise they were regarded as non-consumers.

Knowledge about MS was assessed using questions consisting of 10-items, and women with knowledge scores equal to or above the mean score were classified as having good knowledge, and those who scored below the mean score were classified as having poor knowledge.

### Measurement

Women’s nutritional status was assessed using the mid-upper arm circumference (MUAC). This was measured using a flexible, non-stretchable standard tape at the mid-point between the tips of the ulna and the acromion and olecranon processes on the shoulder blade. The MUAC measurement was made on the right arm to the nearest 0.1 cm. Women with MUAC ≥23 centimeters were regarded as well nourished, and those with MUAC <23 centimeters were regarded as undernourished (7).

Minimum dietary diversity (MDD-W): Data were collected using a 24-h dietary recall method according to Food and Agriculture Organizations’ (FAO) 2016 guideline. For each of the 10 food groups, a woman was asked what they ate 24 h before the data collection time with a score of 1 for yes and 0 for no. Scores were calculated by counting the number of food groups. Finally, women with a score of 5 or more out of 10 were categorized as having adequate dietary diversity; otherwise they were regarded as having inadequate dietary diversity (50).

Pregnancy was determined based on women’s self-report of the first day of their last menstrual period (LMP), and gestational age was calculated based on the LMP and the date of data collection (51).

### Data collection instrument

A pre-tested, interviewer-administered, and structured questionnaire, developed in English, and translated into the local language was used to collect data. Diet-related factors were assessed using a validated 24-h food frequency questionnaire (50). Household socioeconomic status was assessed by permanent household assets using a questionnaire adapted from the Ethiopian demographic and health survey household wealth index assessment tool (5).

### Data collection techniques

Data were collected through face-to-face interview using a mobile phone-based application that allows filling of information electronically in both online and offline means and transfer of data to an online server created for this purpose (52). Ten nurses and two public health professionals were assigned to collect and supervise the data, respectively. Before data collection, the data collection teams were trained for 2 days on interview techniques and questionnaire content.

#### Data processing and analysis

Data were checked daily online for completeness, consistency, and missing values. Once data collection was completed, the raw data were downloaded and exported to STATA statistical software version 14.0 for analysis. Principal component analysis (PCA) was conducted to determine participants’ wealth status and assess women’s knowledge on the importance of consuming fresh MS leaves. Three of the knowledge items [“Can consuming M prevent malnutrition?” “Is MS used as medicine?,” and “Does MS increase breast milk production?”] were removed because of commonalities less than 0.5 (0.49, 0.44, and 0.467, respectively). PCA revealed three factors explaining 85.3% of the total variance, and the factor score of the first factor explaining the maximum variance was used to classify women’s knowledge on the importance of consuming fresh MS leaves. Reliability analysis also showed acceptable internal consistency (Cronbach’s alpha = 0.85). Descriptive statistics including frequencies, percentages, means, and standard deviations were used to describe the characteristics of the study participants. For all explanatory variables, bivariate analysis was performed to assess the presence or absence of association with the outcome variable, and variables with *p*-values less than 0.25 in the bivariate analysis were included in the multivariable logistic regression analysis model. Adjusted odds ratio with its 95% CI and *p*-values < 0.05, respectively, were used to determine the degree of association and statistical significance. The results are presented in tables, figures, and texts.

### Ethics approval and consent to participate

Ethical approval and permission were granted by the Institutional Review Board of Jimma University Institute of Health (reference number: THRPG 1/469/2022). Written approval was obtained from the Gamo zone health department, and health offices of Arba Minch zuria and Chencha districts. Before participation, all participants provided written informed consent as approved by the ethics committee, and all procedures were performed in accordance with the relevant guidelines and regulations of the Declaration of Helsinki on ethical principles for medical research involving human subjects. Data collectors obtained written consent by reading the consent form to participants before the interview and proceeded to interview only after confirming that women were willing to participate in the study. We tried to reduce social desirability bias by conducting interviews with participants in a private place at their homes while at the same time ensuring protection of participants’ privacy, and confidentiality throughout the data collection process.

## Results

### Socio-demographic and economic characteristics of the study participants

A total of 623 pregnant women participated in the study giving a response rate of 98.3%. The mean age the participants was 25 years (SD ± 0.17), 500 (80.26%) of the participants were formally unemployed women who were housewives. Over half (51.69%) and two-third (68.86%) respectively were from rural areas and had less than five family members (Table 1).

**Table 1:** Socio-demographic characteristics of pregnant women in Gamo zone, Southern Ethiopia, 2022.

Variables	Frequency	Percent
**Age of respondent**		
Below 24	273	43.82
24 to 34	328	52.65
Above 34	22	3.53
**Place of residence**		
Rural	322	51.69
Urban	301	48.31
**Religion**		
Muslim	02	0.32
Orthodox	138	22.15
Protestant	483	77.53
**Marital status**		
Married	621	99.68
Other	02	0.32
**Maternal educational level**		
No formal education	119	19.10
Grade 1 to 4	112	17.98
Grade 5 to 8	159	25.52
Grade 9 and10	146	23.43
Grade 11 and 12	34	5.46
College and above	53	8.51
**Husband educational level**		
No formal education	85	13.69
Grade 1 to 4	251	40.42
Grade 5 to 8	221	35.59
Grade 9 and 10	64	10.31
Grade 11 and 12	85	13.69
College and above	251	40.42
**Occupation of the women**		
Governmental worker	24	3.85
Merchant	82	13.16
Student/Farmer	17	2.73
House wife	500	80.26
**Family size**		
Less 5	429	68.86
5 and more	194	31.14
**House hold head**		
Male	597	95.83
Female	26	4.17
**Wealth index**		
Low	199	31.94
Middle	201	32.26
High	223	35.79

### Health and obstetric related characteristics of the study participant

Most of the study participants had less than five pregnancies (91.81%), and deliveries (93.54%).Nearly three-fourth of the participants (72.39%) had history of antenatal attendance (Table 2).

**Table 2:** Obstetrics and health-related characteristics of pregnant women in Gamo zone, southern Ethiopia, 2022.

Variables	Frequency	Percent
**Age at first pregnancy**		
Less or equal to 18 years 19 to 24 years More than 24 years	110 416 97	17.66 66.77 15.57
**Number of pregnancy**		
One to four Five and more	572 51	91.81 8.19
**Number of delivery**		
One to four Five and more	391 27	93.54 6.46
**History of abortion**		
No Yes	546 77	87.64 12.36
**Number of under 5 children**		
One Two	252 135	65.12 34.88
**Pregnancy status**		
Unplanned Planned	210 413	33.71 66.29
**ANC visit**		
No Yes	172 451	27.61 72.39
**GA at first ANC visit**		
Less than 16wks More than or equal to 16wks	220 231	48.78 51.22
**Had Nausea/ vomiting**		
No Yes	294 329	47.19 52.81
**Pre- pregnancy contraceptive use**		
No Yes	293 330	47.03 52.97
**Had health insurance**		
No Yes	352 271	56.50 43.50
**Distance to nearest health center**		
Less than 5 km More than 5 km	470 153	75.44 24.56

### Dietary related characteristics of the study participants

Three hundred nine (49.60, 95% CI, 45.67, 53.52%) of the pregnant women consumed fresh MS leaves in the form of “Kurkufa,” ‘fossosie’, and ‘kita” along with ‘haleko’, a local dish made from fresh MS leaves. Nearly one-quarter of the pregnant women (23.27%) were malnourished, and nearly half (46.07%) did not eat the recommended number of meals during this pregnancy. Only 13 participants (2.09%) skipped meals during the current pregnancy (Table 3).

**Table 3:** Dietary related characteristics of pregnant women in Gamo zone, Southern Ethiopia, 2022.

Variables	Frequency	Percent
**Maternal nutritional status**		
Under nourished	145	23.27
Well nourished	478	76.73
**Meal frequency per day**		
Two	13	2.09
Three	274	43.98
Four	249	39.97
Five	87	13.96
**Increased the frequency and amount of dietary intake**		
No	393	63.08
Yes	230	36.92
**Dietary diversity for women**		
No	347	55.70
Yes	276	44.30
**Food aversion**		
No	566	90.85
Yes	57	9.15
**Getting nutritional counseling**		
No	353	56.66
Yes	270	43.34
**Fresh moringa leaf consumption**		
Not consumed	314	50.40
Consumed	309	49.60

#### Women’s knowledge on moringa stenopetala leaf consumption

**Figure 1:**
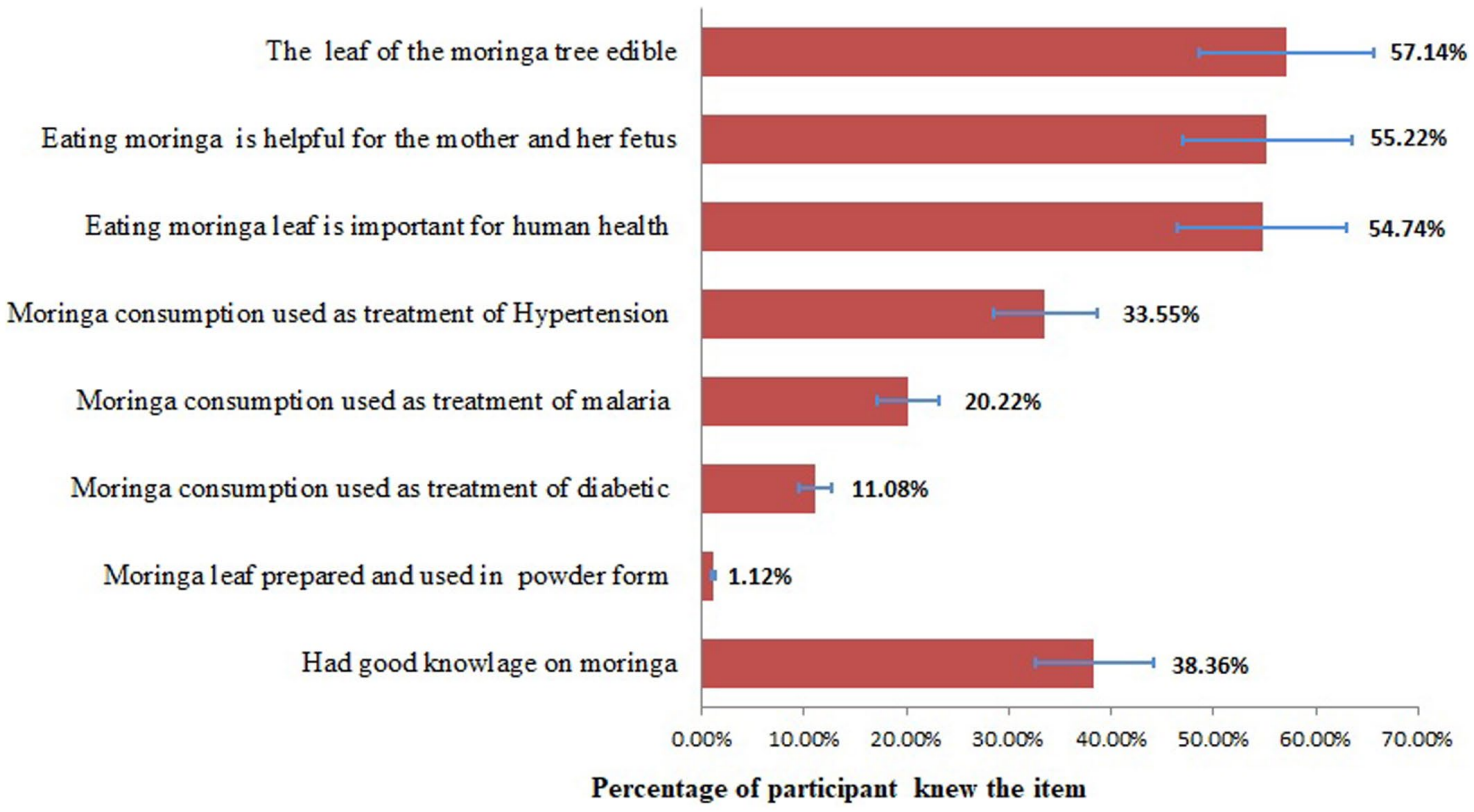
Knowledge of pregnant women on the importance of moringa in Gamo zone, Southern Ethiopia, 2022.

Two hundred thirty-nine (38.36%) of the study participants had good knowledge about the importance of consuming fresh MS leaves. Participants were most likely to know about the edible parts of the MS tree (57.14%), followed by the importance of consuming MS for the pregnant mother and her fetus (55.22%). However, what they least knew was that MS can be consumed in powder form (1.12%), followed by its use in treating diabetes (11.08%) (Figure 1).

### Frequency of fresh moringa stenopetala leaf consumption

**Figure 2:**
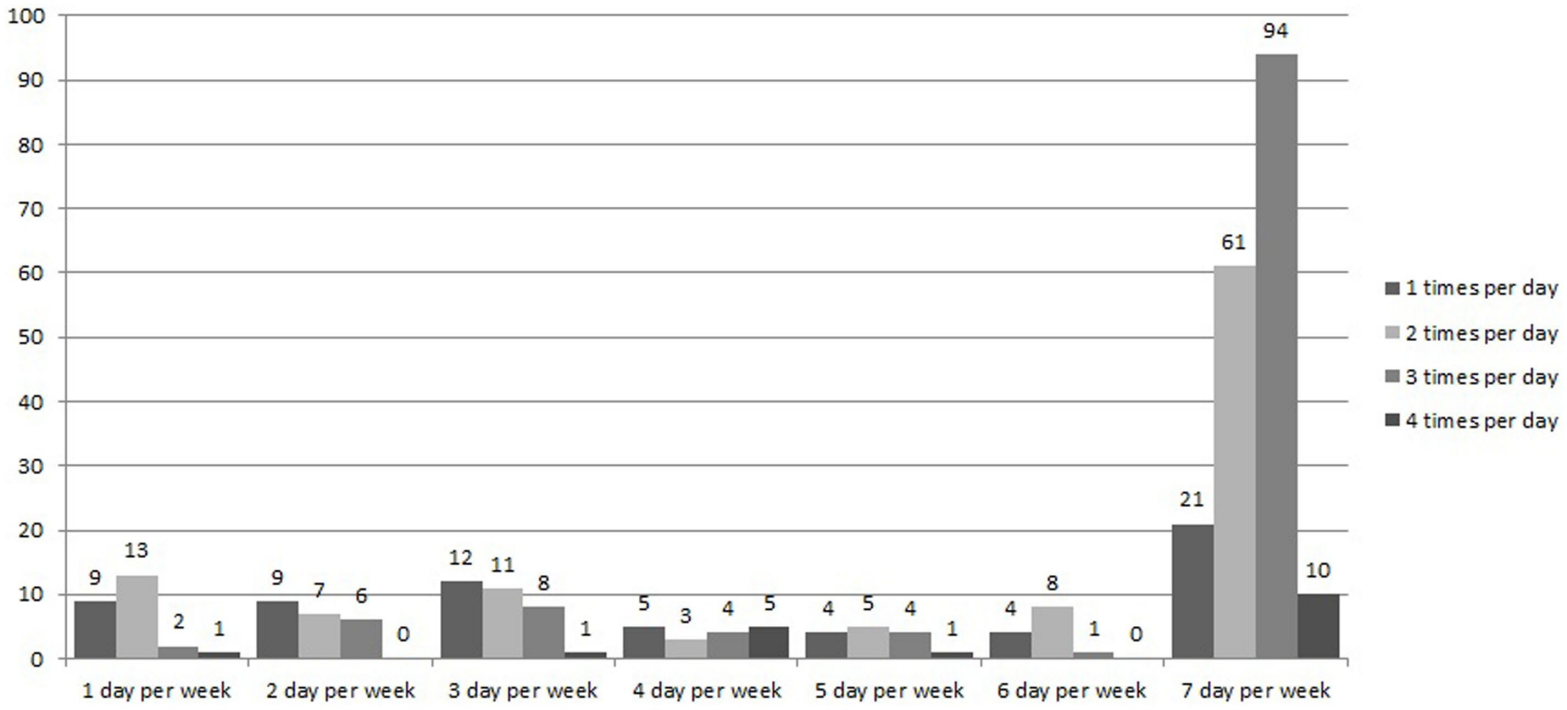
Frequency of fresh moringa stenopetala leaves consumption among pregnant women in Gamo zone, Southern Ethiopia, 2022.

Approximately half of women who consumed fresh MS leaves (44.34%) consumed it three or more times a day, and 178 women (77.4%) used it as a food source (Figure 2).

#### Factors associated with fresh moringa stenopetala leaf consumption

Of the 12 candidate variables for multivariable logistic regression analysis, seven associated with MS leaf intake during pregnancy. Age, place of residence, attendance to antenatal care, use of contraception before pregnancy, increased number of frequency, and amount of food intake, consumption of five or more food groups within the past 24 h, and women’s knowledge of the importance of consuming fresh MS leaves during pregnancy. The odds of fresh MS leaves intake was higher in women aged <24 years [AOR = 2.92, 95%CI (1.51, 5.63)], and in rural residents [AOR = 1.97, 95% CI (1.10, 3.50)] compared with their age and residence counterparts, respectively. Visited health care facility for ANC [AOR = 2.08, 95% CI (1.03, 4.21)], increased the frequency and quantity of food intake during pregnancy [AOR = 2.89, 95% CI (1.54, 5.41)], consumed five or more food groups in the last 24 h [AOR = 2.33, 95% CI (1.33, 4.08)], using contraceptives before pregnancy [AOR = 1.88, 95% CI (1.03, 3.55)], and had good knowledge on the importance of MS consumption [AOR = 9.76, 95% CI (5.30, 17.95)] were positively associated factors (Table 4).

**Table 4:** Factors associated with fresh moringa leaf consumption during pregnancy in Gamo zone, southern Ethiopia, 2022.

Variable	Consumer *n* = 309	Non-consumer *n* = 314	COR (95% CI)	AOR (95% CI)
**Age in years**				
Less 24	166 (53.72)	107 (34.08)	2.19 (1.58, 3.04)**	2.92 (1.51, 5.63)**
24 to 34	136 (44.01)	192 (61.15)	1	1
Above 34	7 (2.27)	15 (4.78)	0.66 (0.26,1.66)	1.02 (0.28, 3.76)
**Place of residence**				
Rural	185 (59.87)	137 (43.63)	1.93 (1.40, 2.65)**	1.97 (1.10, 3.50)*
Urban	124 (40.13)	177 (56.37)	1	1
**ANC follow up**				
No	57 (18.45)	115 (36.62)	1	1
Yes	252 (81.55)	199 (63.38)	2.55 (1.76, 3.69)**	2.08 (1.03, 4.21)*
**Pre- contraceptive use**				
No	126 (40.78)	167 (53.18)	1	1
Yes	183 (59.22)	147 (46.82)	1.65 (1.20, 2.26)**	1.88 (1.03, 3.55)*
**Increased the frequency and amount of dietary intake**				
No	138 (44.66)	255 (81.21)	1	1
Yes	171 (55.34)	59(18.79)	5.35 (3.73, 7.68)**	2.89 (1.54, 5.41)**
**Dietary diversity**				
No	139 (44.98)	208 (66.24)	1	1
Yes	170 (55.02)	106 (33.76)	2.40 (1.73, 3.31)**	2.33 (1.33, 4.08)**
**Knowledge on moringa**				
Poor knowledge	113 (36.57)	271 (86.31)	1	1
Good knowledge	196 (63.43)	43 (13.69)	10.93 (7.35,16.24)**	9.76 (5.30, 17.95)**

** *p* < 0.01; *p-value <0.05.

## Discussion

Pregnancy is a critical period during which significant physiological and biochemical changes occur in the mother and the developing fetus (53). Maternal diet before and during pregnancy is a key factor in the health of the mother as well as the child and can have a lasting impact on the child’s health and future development. MS is a nutritious plant grown in tropical regions of the developing world. All parts of the tree can be used in a variety of beneficial ways, but the leaves are a particularly good source of vitamins and other nutrients. Gram for gram, fresh moringa leaves have been reported to contain seven times more vitamin C than oranges, four times more vitamin A than carrots, three times more iron than spinach, four times more calcium than milk, and three times more vitamin D than cereals. They also contain more potassium than bananas and twice as much protein as yogurt (35). Micronutrient and vitamin intake is an important measure to promote maternal and child nutrition, health, and well-being, and should be continued throughout pregnancy, regardless of the nutritional status of the mother, especially in low- and middle-income countries. Moringa stenopetala leaves contain both micronutrients and vitamins, but consumption is still low in Ethiopia. In this study, we attempted to investigate the intake of fresh MS leaves and factors associated with the consumption among pregnant women in the Gamo zone of southern Ethiopia. The intake rate of fresh MS leaves among pregnant women was 49.60%. This is higher than previous studies in adults in central Ethiopia and Africa (39, 42), but lower than studies in Mauritius, Togo, and India (39, 40, 44). A possible explanation for this difference is probably the difference in culture, food habits, and awareness of the study participants. Pregnant women under 24 years of age were three times more likely to consume fresh MS leaves than those aged 24–34 years. This may be due to globalization, increased access to various media (54), and “education for all” (55) helping to improve dietary habits among young people. This contradicts a study conducted in Mauritius (44). This difference may be due to differences in age and food culture. Our study shows that rural residents are twice more likely to consume fresh MS leaves than urban residents. Similar results were reported in a study in Mauritius (44). A possible reason for this is the ease of growing and obtaining the crop in rural areas, increasing the likelihood of consuming fresh MS. Furthermore, rural residents are less likely to utilize modern healthcare due to lack of awareness and accessibility. Thus, they might rely on traditional medicine which promotes the consumption of MS leaves for its medicinal benefits (56). Pregnant women who visited health facilities for ANC were twice as likely to eat fresh MS leaves compared to non-visitors. The fact that ANC service is a good opportunity to provide pregnant women with advice on proper dietary habits (57) might increase their knowledge on healthy diet during pregnancy, which acts as a fuel for the intake of nutritious foods such as MS (58, 59).

Participants who increased their food intake during pregnancy were three times more likely to eat fresh MS leaves compared to participants who did not increase their food intake during pregnancy. Similarly, women who consumed five or more food groups were approximately twice as likely to consume fresh MS leaves compared to their counterparts. This can be explained by the fact that an increase in the frequency and amount of food intake can be used to increase the possibility of dietary diversity (58, 60). This could be because an increase in the frequency, amount, and food groups of meals could contribute to an increase in the consumption of more diverse foods including fresh MS leaves. Pregnant women with good knowledge about the importance of MS leaves were 10 times more likely to eat MS leaves compared to those with a lack of knowledge. This is in line with studies conducted in Mauritius (44) and Ethiopia (61) and could be because basic knowledge about nutrition can guide food choices (62). Women who used contraceptives before pregnancy were twice as likely to consume fresh MS leaves compared to those who did not. This may be because extended stays in health facilities promote health-promoting behaviors and the consumption of various food groups, increasing the likelihood of consuming fresh MS leaves (58). More than half of the pregnant women in the study area did not eat fresh MS leaves during pregnancy, and the majority of them did not have sufficient knowledge about the importance of MS leaves. The implication of these findings is that improved knowledge about the importance of fresh MS leaves consumption during pregnancy through increased utilization of health services, nutritional advice, and information dissemination could increase the consumption of fresh MS leaves, thereby allowing pregnant women to benefit from the nutritional content of MS.

### Strength and limitations

The study was conducted at the community level in home gardens that provide an amenity to address a sensitive issue and is intended to be generalizable. There is a chance of recall bias on responses about dietary diversity and MS leaves consumption patterns particularly because of our participants background of education and culture. However, we have considered data from the most recent month before the survey period which is deemed to minimize recall bias regarding consumption habits. Added on this, the cross-sectional nature of the data might affect temporality and strength of the evidence reported from this study (63).

## Conclusion

The study found that only half of the pregnant women consumed fresh MS leaves during pregnancy. Age, place of residence, utilization of health services (ANC visits and contraceptive use), knowledge about the importance of consuming MS leaves, higher frequency and amount of food intake, and consumption of more than five food groups were independently associated with consumption of fresh MS leaves. Therefore, the relevant authorities should promote the utilization of this wonderful gift to increase knowledge and consumption habits. Furthermore, the above factors associated with MS leaf consumption should be taken into consideration in the efforts. Policymakers should invest more and pay attention to the production of “high quality” food from plants like MS. Further research is needed to determine the amount of fresh MS leaves consumed per meal (per serving) and other relevant factors that may prevent this group from consuming this wonderful plant.

## Data availability statement

The raw data supporting the conclusions of this article will be made available by the authors, without undue reservation.

## Ethics statement

The studies involving humans were approved by the Institutional Review Board (of) Institute of Health, Jimma University, with reference number Ref. No. THRPG 1/469/2022. The studies were conducted in accordance with the local legislation and institutional requirements. The participants provided their written informed consent to participate in this study.

## Author contributions

ZD: Conceptualization, Data curation, Formal analysis, Funding acquisition, Investigation, Methodology, Project administration, Resources, Software, Supervision, Validation, Visualization, Writing – original draft, Writing – review & editing. GD: Conceptualization, Data curation, Formal analysis, Funding acquisition, Investigation, Methodology, Project administration, Resources, Software, Supervision, Validation, Visualization, Writing – review & editing.
